# Mitochondrial DNA is unsuitable to test for isolation by distance

**DOI:** 10.1038/s41598-018-25138-9

**Published:** 2018-05-31

**Authors:** Peter R. Teske, Tirupathi Rao Golla, Jonathan Sandoval-Castillo, Arsalan Emami-Khoyi, Carl D. van der Lingen, Sophie von der Heyden, Brent Chiazzari, Bettine Jansen van Vuuren, Luciano B. Beheregaray

**Affiliations:** 1 Centre for Ecological Genomics and Wildlife Conservation, Department of Zoology, University of Johannesburg, Auckland Park, 2006 South Africa; 2 Molecular Ecology Lab, College of Science and Engineering, Flinders University, Adelaide, SA 5001 Australia; 3 Branch: Fisheries Management, Department of Agriculture, Forestry and Fisheries, Private Bag X2, Vlaeberg, 8012 South Africa; 4 Department of Biological Sciences and Marine Research Institute, University of Cape Town, Private Bag X3, Rondebosch, 7700 South Africa; 5 Evolutionary Genomics Group, Department of Botany and Zoology, University of Stellenbosch, Private Bag X1, 7602 Matieland, South Africa; 6 School of Life Sciences, University of KwaZulu-Natal, Westville, Durban, 4001 South Africa

## Abstract

Tests for isolation by distance (IBD) are the most commonly used method of assessing spatial genetic structure. Many studies have exclusively used mitochondrial DNA (mtDNA) sequences to test for IBD, but this marker is often in conflict with multilocus markers. Here, we report a review of the literature on IBD, with the aims of determining (a) whether significant IBD is primarily a result of lumping spatially discrete populations, and (b) whether microsatellite datasets are more likely to detect IBD when mtDNA does not. We also provide empirical data from four species in which mtDNA failed to detect IBD by comparing these with microsatellite and SNP data. Our results confirm that IBD is mostly found when distinct regional populations are pooled, and this trend disappears when each is analysed separately. Discrepancies between markers were found in almost half of the studies reviewed, and microsatellites were more likely to detect IBD when mtDNA did not. Our empirical data rejected the lack of IBD in the four species studied, and support for IBD was particularly strong for the SNP data. We conclude that mtDNA sequence data are often not suitable to test for IBD, and can be misleading about species’ true dispersal potential. The observed failure of mtDNA to reliably detect IBD, in addition to being a single-locus marker, is likely a result of a selection-driven reduction in genetic diversity obscuring spatial genetic differentiation.

## Introduction

Species with wide distributions are rarely completely panmictic^1^, even in potentially open systems such as the world’s oceans^2,3^. When individual dispersal distances are smaller than a species’ range, genetic drift acting on neutral genetic markers will over time result in a positive correlation between genetic differentiation between locations and the geographic distance separating them, a pattern known as “isolation by distance” (IBD)^4,5^. IBD is currently the most frequently tested hypothesis in studies of spatial genetic structure, and is considered to provide a suitable means of indirectly estimating a species’ dispersal ability^6,7^. The method is also considered important in the management of exploited species. For example, in high-dispersal marine species in which the existence of clear stock boundaries is unlikely, tests for IBD can help to define regional geographic units that respond to exploitation relatively independently^8^.

Despite their popularity, tests for IBD are often criticised. One recent debate concerns the use of Mantel tests in assessing relationships between genetic and spatial data^9^. It has been suggested that the power of other approaches (e.g. linear correlation, regression and canonical analysis) is far greater than that of Mantel tests in detecting a relationship when one is present^10^. Another problem, and one that affects IBD *per se* rather than merely the way in which it is identified, concerns its use under inappropriate conditions. Most prevalently, IBD can be strongly affected by the existence of distinct regional genetic clusters^11^. For example, secondary contact between populations that have been isolated from each other in glacial refugia^12^ or the existence of sister lineages in adjacent biogeographical regions^13^, will give the impression of limited dispersal ability even when each population is genetically homogeneous within its range. The fact that some regional genetic clusters display physiological adaptations to the region they occupy, and some are morphologically distinguishable from each other^14,15^ suggests that significant IBD may often be the result of inappropriately lumping distinct cryptic species. When these regional units are analysed individually^16–20^ or when stratified Mantel tests are applied and locations of populations are permuted within clusters^21^, the pattern of IBD is often no longer found, and the species’ true dispersal potential is revealed. Nonetheless, due to their simplicity and the fact that geographic distance can serve as a proxy for numerous other factors that limit gene flow between locations, tests for IBD will likely continue to be used as a standard method of assessing a species’ dispersal ability. As such, they represent a simple baseline method against which to test more complex landscape genetic approaches^22^.

One aspect of IBD that has received little prior attention is the question which genetic markers are most suitable to detect it, and under which conditions. Numerous studies have dealt with the fact that the power of different genetic markers to detect genetic structure can differ considerably, and may result in conflicting findings^23,24^, but the implications of this on tests for IBD remain poorly explored. With the increased application of next-generation sequencing approaches in species-level studies, the information content of the markers that can be used for IBD has increased by several orders of magnitude. It is thus timely to determine how traditional markers such as single-locus mtDNA sequence data or a limited number of microsatellite loci perform relative to the much larger SNP data sets.

In the present paper, we searched the literature to get an overview of the performance of mtDNA compared to other markers. An *ad-hoc* Google Scholar search using the search terms *DNA barcoding*, *isolation by distance* and *mitochondria* returned 17 800 hits. Although not all these studies would have conducted tests for IBD using this single locus, the result suggests that the use of mtDNA in tests for IBD is very common, and, despite the existence of more powerful alternatives, remains a standard method to assess species’ dispersal potential. We then compared microsatellite and selectively neutral single nucleotide polymorphism (SNP) datasets generated using double-digest restriction site associated DNA sequencing (ddRADseq, i.e. a reduced representation genotyping method employing high-throughput sequencing^25^) with mtDNA data in four cases where no IBD was found with the latter marker. The data sets used in this study originate from widespread marine species with high dispersal ability (long-lived planktonic larvae and/or active adult dispersal) along continuous coastlines. The finding that the alternative markers consistently found strong evidence for IBD when none was found with mtDNA when accounting for the existence of spatially distinct population clusters suggests that the exclusive use of the latter can result in questionable conclusions about species’ dispersal ability.

## Materials and Methods

### Literature review

To gain an overview about the performance of mtDNA in identifying IBD, we searched for (a) studies that used this marker to test for IBD along the South African coastline (where the ranges of the evolutionary lineages of high-dispersal coastal species tend to be strongly linked to the region’s temperature-defined marine biogeographic provinces^13,26^), and along the coast of temperate southern Australia. The aim was to determine whether significant IBD is mostly the result of inappropriately pooling distinct regional genetic clusters (*sensu* Meirmans *et al*.^11^). We only included species with high dispersal potential (i.e. those with active adult dispersal and/or planktonic larvae) because in low-dispersal species (i.e. whose offspring remains in the parent habitat) genetic structure is often not clearly linked with contemporary biogeography^13,27^. We also searched for (b) studies that compared IBD between mtDNA and microsatellites. For (b), various combinations of the following search terms were used in Google Scholar to identify suitable papers: *isolation by distance* (or *isolation by geographic distance*), *IBD*, *mitochondria* (or *mtDNA*), *discrepancy*, *contrasting*, *conflicting*, *microsatellite*. A table was then constructed for each, and general trends discussed.

### Acquisition and generation of genetic data

**Figure 1:**
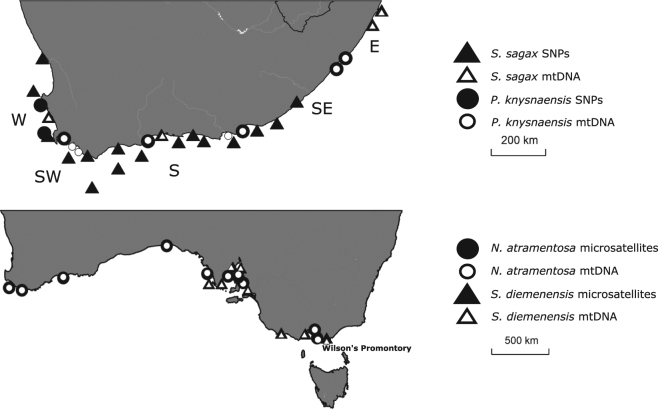
Sampling sites in South Africa (top) and temperate southern Australia (bottom). In both figures, small white symbols indicate mtDNA data and large black symbols represent either microsatellites or SNPs from ddRADseq. Marine bioregions in South Africa are indicated as W (west coast), SW (south-west coast), S (south coast), SE (south-east coast) and E (east coast); in Australia, distinct bioregions are separated by a biogeographic barrier at Wilson’s Promontory.

We compared IBD in four marine species that occur along continuous marine regions in temperate southern Australia and South Africa. These include two Australian gastropods (the snail *Nerita atramentosa* and the limpet *Siphonaria diemenensis*), and two South African teleosts (the goby *Psammogobius knysnaensis* and the sardine *Sardinops sagax*). These species were selected because (a) comprehensive range-wide genetic data sets were available and (b) none exhibits mtDNA-based genetic structure, even though co-distributed species with similar dispersal potential may comprise multiple regional evolutionary lineages^26,28^. The mtDNA data were obtained from the following studies: *N. atramentosa* and *S. diemenensis*^26^; *P. knysnaensis*^29^; *S. sagax*: B. Chiazzari, unpubl. data. Data from other markers included microsatellites for the Australian gastropods (*N. atramentosa*^30^; *S. diemenensis*^31^), and SNP data for the South African teleosts. The latter were generated for the present study. Briefly, DNA was extracted from fin clip or muscle tissue using the cetyltrimethylammonium bromide (CTAB) method^32^. Double-digest restriction site associated DNA sequencing (ddRADseq) was performed at the Molecular Ecology Laboratory at Flinders University following the protocol of ddRADseq.^25^ with restriction enzymes SbfI and MseI and modifications as described in Brauer *et al*.^33^. Libraries were sequenced on an Illumina HiSeq 2000 platform at the McGill University and Genome Québec Innovation Centre. BayeScan v2.0^34^ was then used to identify and exclude loci potentially under selection on the basis of having significantly elevated allele frequency differences between populations. We specified default settings and a false discovery rate of 5%. In order to create data sets comprising selectively neutral loci, we excluded 203 outlier SNPs identified with BayeScan from the total sardine data set (out of a total of 11649 SNPs) and 572 outlier SNPs from the goby data set (out of a total of 8543 SNPs), and used only the remaining loci to test for IBD. Sample sites for all four species are shown in Fig. 1 (see Supplementary Table S1 for details concerning sampling sites and number of samples). Ethics clearance for the sample acquisition of the two teleosts was granted by the ethics committee of the University of Johannesburg. All experiments were performed in accordance with relevant named guidelines and regulations.

### Isolation by geographical distance

Genetic distance matrices were created in Arlequin v3.5.2.2^35^ that comprised *F*-statistics^36^ between pairs of sites, using Φ_ST_ for mtDNA data and *F*_ST_^36^ for microsatellite and SNP data. The most suitable genetic distance model for each mtDNA data set was determined using the Bayesian Inference Criterion^37^ in MEGA v.7.0.26^38^ and were the Kimura 2-Parameter model^39^ for *Sardinops sagax* and *Psammogobius knysnaensis*, the Tamura-Nei model^40^ for *Nerita atramentosa*, and the Tamura 3-Parameter model^41^ for *Siphonaria diemenensis*. Geographical distances between pairs of sites were measured as the shortest along-coast distances using the path tool in Google Earth Pro v 7.3.0.3832.

Relationships between genetic and geographic distance matrices were calculated using (a) a Mantel test^9^ and, to account for possible type II error^10^ (b) simple linear regression. The Mantel tests were performed with the R package adegenet 1.4–0^42^. This program tests for significant IBD by comparing the observed correlation with a histogram of simulated correlation categories and their frequency under the assumption of no IBD. We selected Spearman rank correlations^43^ and 999 matrix permutations. The program also creates scatterplots of geographic vs. genetic distance, that depict the density between points. Densities between points in a scatterplot can be used to determine whether the data originated from a single genetic cline or from two or more distinct regional clusters for each of which the test for IBD should be performed separately^11^. To confirm that the neutral SNP data did not comprise multiple genetic clusters, the k-means clustering algorithm in adegenet was used to identify the number of clusters that maximise between-cluster variation. Simple linear regression to corroborate the results of the Mantel tests was performed in SigmaStat 1.0 (Systat Software, San Jose, CA).

### Data availability

The datasets generated and/or analysed during the current study are available from the corresponding author on reasonable request.

## Results

### Tests for IBD: literature review of patterns in two coastal regions

**Figure 2:**
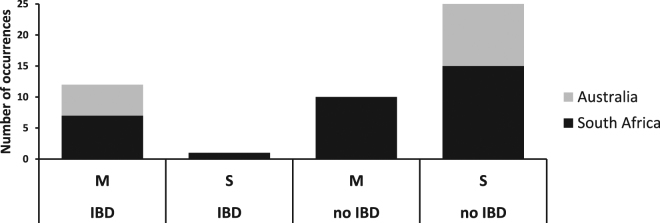
Number of instances of significant IBD and no IBD being found in South African (black) and temperate southern Australian (grey) coastal marine species when samples were collected across multiple marine biogeographical provinces (M) or within single provinces (S).

A synthesis of studies on the South African and southern Australian coastlines indicated that significant IBD was found much more frequently when these tests were performed on data sets that originated from multiple (M) biogeographical regions than when tests were performed within single regions (S) (Fig. 2, Supplementary Table S2). This trend was particularly clear in Australia, even though the fact that most studies employed microsatellites would intuitively suggest that the chances of finding significant IBD within provinces would be greater. A single study reported significant IBD within a single province (*Parechinus angulosus*, based on mtDNA data), but this result was considered unreliable because of a small sample size^20^.

Particularly clear examples of significant IBD that was no longer found when data from individual biogeographical regions were analysed separately include *Acanthochiton garnoti, Cyclograpsus puncatus, Oxystele tigrina, O. variegata*^20^ and *Tetraclita serrata*^44^ in South Africa, as well as *Donax deltoides*^45^ and *Haliotis rubra*^46^ in Australia.

### Test for IBD: literature review of marker types

**Figure 3:**
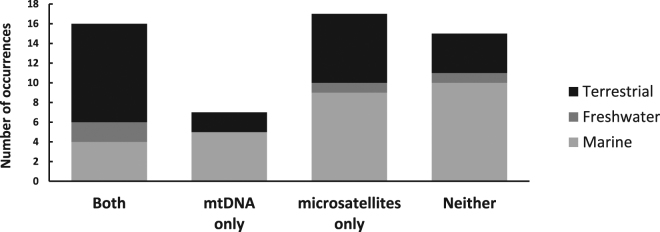
Number of studies that used both mtDNA and microsatellites to test for IBD in which significant IBD was found for both, either or neither marker. For each category, the habitat through which a particular taxon is most likely to disperse is indicated.

A comparison of IBD between mtDNA and microsatellites in studies that used both markers revealed that marker-specific discrepancies are common, and were found in almost half of the studies reviewed (Fig. 3, Supplementary Table S3). Microsatellites were more likely to identify significant IBD when mtDNA did not than the inverse, and there was no clear indication that this trend is habitat-specific.

### Tests for Isolation by Distance: this study

**Figure 4:**
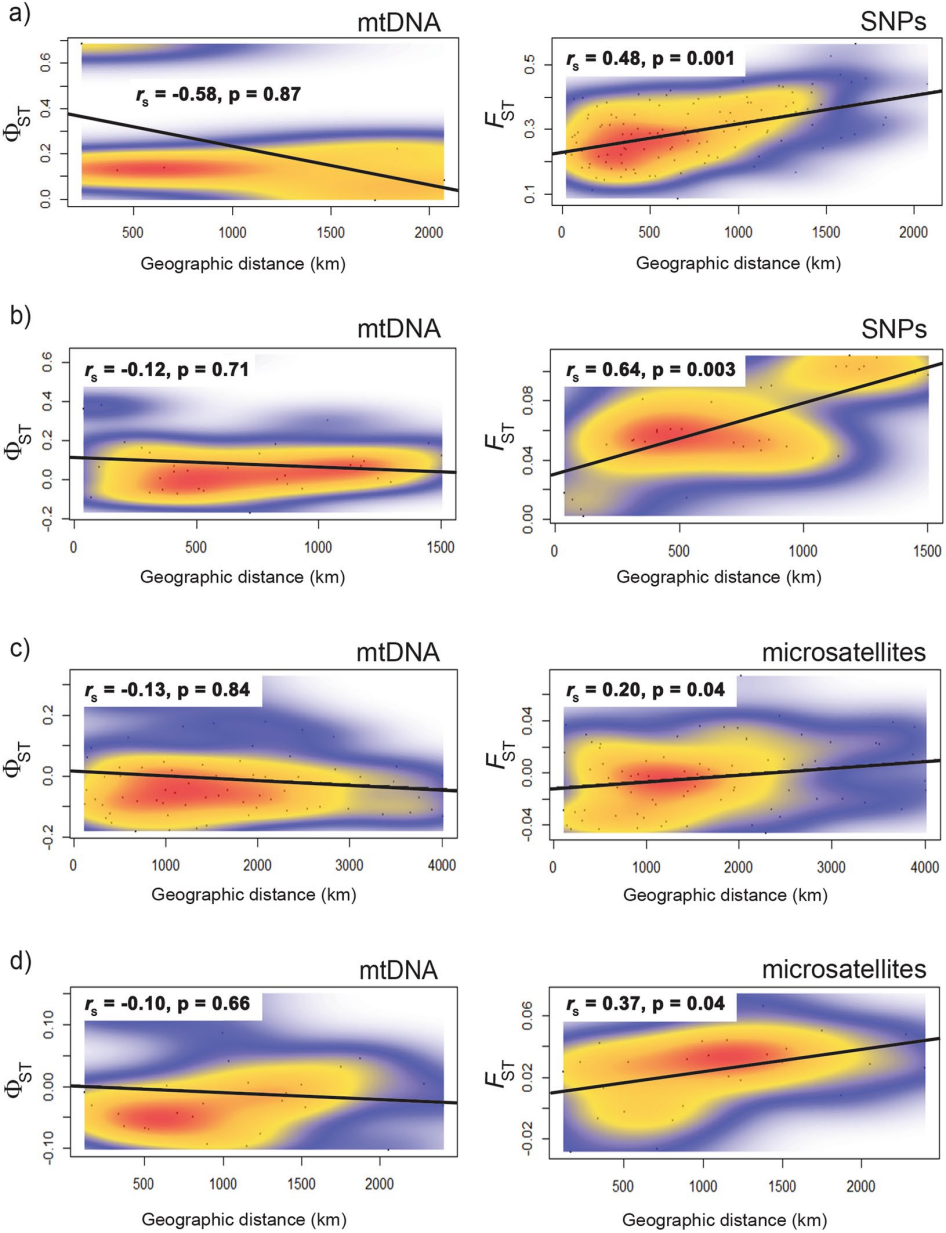
Plots of geographic distances vs. *F*-statistics for the following species (plots on the left show mtDNA data, those on the right SNP or microsatellite data): (**a**) *Sardinops sagax*; (**b**) *Psammogobius knysnaensis*; (**c**) *Nerita atramentosa*; (**d**) *Siphonaria diemenensis*. The density of data points is indicated by colours.

Mantel tests performed on geographic distance matrices and matrices comprising pairwise *F*-statistics between sites identified no IBD for any of the mtDNA data sets (Fig. 4, left panels); all correlations were negative, which indicates more differentiation within sites than between pairs of sites. In contrast, all correlations were positive for the nuclear markers (right panels) and Mantel tests were significant. Scatter plots with information on point density showed no strong evidence for the existence of multiple regional clusters for any species or marker, and k-means clustering confirmed that both SNP data sets comprised single genetic clusters (Supplementary Fig. S1), information that was already available for the other data sets^26,29^.

The Mantel test results were confirmed by linear regression analysis, with the exception of the microsatellite data of *N. atramentosa*, where significant IBD was no longer found (Supplementary Table S4). The statistical power (i.e. the probability of correctly rejecting the null hypothesis of no IBD when IBD is present) of all non-significant tests was below the desired power of 0.8^47^, indicating that these data sets were not sufficiently informative to identify IBD, and failure to identify it thus needs to be interpreted with caution. The power of the tests performed on the two SNP data sets was highest (both 1.0, i.e. the maximum possible).

As IBD analyses can be affected by the number of sites^22^, we reanalysed the sardine SNP data set (which included many sites for which no COI data were available, Supplementary Table S1) by including only four sites that were represented in both data sets. Both the Mantel test and the linear regression analyses remained signficant (Mantel *r*_s_ = 0.83, P = 0.035; Linear regression *R*^2^ = 0.8, *F* = 32.0, P < 0.001), and the power of the latter was only slightly below that of the complete data set (0.97).

## Discussion

Tests for isolation by distance that employed mtDNA sequence data became popular in the 1990s, when this became the marker of choice because of its faster mutation rate and smaller genome size compared to allozymes^48,49^. It has been used rather uncritically ever since, and it is still not fully understood why it is often in conflict with nuclear markers^23^, although the smaller effective population size^50^, which results in a higher rate of lineage sorting^51^, is often cited. It is likely that the increasing popularity of DNA barcoding will result in an increase in the number of studies that will exclusively use mtDNA sequences to test for IBD, as it is now possible to readily apply this approach on entire communities (e.g.^52–54^). For that reason, a critical assessment of the power of mtDNA in detecting IBD is timely.

### mtDNA vs. microsatellites

Our review of studies that used mtDNA together with microsatellites indicated that the two markers were often in conflict, with microsatellites more likely to identify IBD when mtDNA did not. We are aware of no studies that employed power analyses to test for the probability of type II error, but linear regression analyses of our empirical data (Supplementary Table S4) indicate that the power of the mtDNA data sets normally used to test for IBD is often far below the acceptable threshold of 0.8^47^, and non-significant results questionable. It is noteworthy that several additional studies we reviewed reported IBD for only one of these markers even though both were used in other analyses^55–58^, perhaps because the other was in conflict. Intuitively, one would expect a comprehensive data set of hypervariable microsatellites to be more suitable to detect IBD, not only because of their high mutation rate^59,60^ but also because different combinations of alleles arise in each generation and are sorted over geographical space^61^, making microsatellites useful to study spatial patterns at geographic scales as small as tens of meters^62,63^. As such, it is possible that some of the studies in which significant IBD was found with mtDNA data, but not microsatellites, may be a result of the latter employing microsatellite loci with comparatively little variation and/or a small number of loci, in addition to potential technical artefacts such as large allele dropout and null alleles. Any general conclusions about the usefulness of microsatellites to detect IBD is thus hampered by the fact that, unlike mtDNA sequences, the microsatellite data sets used in different studies are not directly comparable. However, a detailed assessment of the factors responsible for this discrepancy is beyond the scope of the present meta-analysis. Our results for the Australian gastropods, which employed highly informative microsatellite data sets suitable to detect self-recruitment^30,31^, and for which the same sites and individuals were used as for the corresponding mtDNA data, represent an important contribution towards facilitating more direct comparisons between the two marker types. In the light of these findings, it is clear that reliable results at a minimum require congruence between multiple marker types.

### Pooling of distinct regional lineages

A review of studies on marine animals that tested for IBD further indicated that in many cases where a significant positive correlation was found with mtDNA data, it could potentially be attributed to the pooling of spatially discrete evolutionary lineages. There was no convincing evidence for significant IBD within single marine biogeographic provinces for any of the markers employed. Temperature is arguably the most important environmental predictor of coastal biogeographic patterns^64^, and in South Africa, many species are subdivided into regional evolutionary lineages whose ranges coincide with temperature-defined biogeography^13^. Physiological data indicate that sister lineages in adjacent marine regions have different thermal tolerance ranges^14,15,65^. Because of this, the pooling of data from multiple lineages does not provide information on species’ dispersal potential *per se*, because it ignores the fact that dispersal of regional evolutionary lineages is constrained by their inability to establish themselves in the habitat of their sister lineage rather than the inability to reach that habitat. The situation is similar in temperate Australia, where species that are present on either side of the marine biogeographic barrier at Wilson’s Promontory (Fig. 1) often comprise distinct southern and eastern lineages that do not readily establish themselves in each other’s habitat^66,67^ and that should be treated as distinct when testing for IBD. Numerous additional examples of distinct evolutionary lineages occurring along continuous coastlines that are separated by biogeographical transition zones have been reported elsewhere, including California and Florida^27^, Chile^68,69^ and various sites along the north-eastern Atlantic and the Mediterranean^70,71^. The above considerations likely apply to all of them.

### A process-driven explanation for the poor performance of mtDNA

Mitochondrial DNA sequence data originate from a single locus and, unlike in microsatellite data, there is thus no differentiation in the frequency of alleles from different loci from one generation to the next that can generate spatial genetic structure. Nonetheless, the high mutation rate of mtDNA compared to nuclear DNA^72^ should theoretically make it suitable to study IBD because of high levels of genetic diversity at the intrapopulation level. When dispersal is limited, the frequency of new mutations that have arisen at a particular location is expected to decrease away from that location. A number of recent studies have rejected the idea that the high mutation rate *per se* results in high genetic variation at or below the species level, and have indicated that it is in fact much lower than expected under the neutral model of evolution^73^. Instead, the maximum genetic diversity (MGD) hypothesis, which assumes a saturation plateau beyond which genetic diversity no longer increases^74,75^ may describe mtDNA evolution more adequately. At the population level, the low level of mtDNA diversity is a result of strong variation-reducing selection that may include strong purifying selection^73,76^ or selective sweeps driven by physiological constraints within environmentally homogeneous regions^77^. Interestingly, this selection not only affects non-synonymous sites but also third character codon positions where mutations do not result in amino acid changes^78–80^. Although there is no consensus, explanations such as linkage of sites affected by synonymous mutations to regions under intense selection^81–84^ or codon biases during translation^78,85,86^ have been invoked. The consequence of such processes (which do not merely drive selection at the population level but even at the individual or organelle level^73^), is that mutations and reversions in mitochondrial genes at the population level are typically limited to relatively few sites, and homoplasies are common^76,87^. In the context of IBD, it is possible that the same mutations arising independently across the physiologically constrained range of a population may obscure already limited neutral genetic differentiation driven by geographic distance. It has been suggested that the combination of variation-reducing selection at the population or species level, and adaptive selection at the between-species level, makes mtDNA particularly powerful for the molecular differentiation of species because it increases the ‘barcoding gap’^76,87^. However, the very departures from the expectations of selective neutrality that are so useful for DNA barcoding may be partly responsible for making this marker unsuitable to detect IBD.

### Relevance of the study findings to non-marine taxa

Many marine species have very large census population sizes^88^. This suggests that for selectively neutral loci, marine populations should also have very high genetic diversity, and this has been confirmed using nuclear DNA^89–92^. However, as explained in the previous paragraph, the assumption of selective neutrality does not apply to mtDNA. A meta-analysis of >1600 animal species^91^ showed that within-species (or population level) mtDNA diversity is very similar across animal phyla, irrespective whether they have large or small census population sizes, or whether they are aquatic or terrestrial. An explanation for the low genetic diversity of large populations is provided by Gillespie’s^77^ ‘genetic draft’ hypothesis, which states that beneficial mutations are more likely to arise in large populations, and these result in recurrent selective sweeps that reduce genetic diversity at the mitogenome level. Low variation in animal mtDNA, irrespective of census population size, range or habitat type has been confirmed by numerous phylogeographic and DNA barcoding studies^93–97^. These considerations indicate that there is little reason to assume that the failure of mtDNA to reliably detect IBD is limited to marine species, and this is confirmed by the fact that of the taxa used for Fig. 3, the proportion of studies that found discrepancies between mtDNA and microsatellites were very similar for different habitat types (39% of terrestrial taxa and 46% of aquatic taxa). As both aquatic and terrestrial species tend to be subdivided into distinct, geographically isolated evolutionary lineages^98^ that may be under divergent selection, the artefact of identifying IBD when distinct populations are pooled shown in Fig. 2 also applies to non-marine animal taxa.

### Application of neutral SNP data

In addition to the issues in identifying IBD that are related to marker type and evolutionary history, the power to detect IBD is also related to the number of populations included in the analysis^7,22^. Together, these factors indicate that a sampling design hampered by insufficient financial commitment, capacity and sampling effort may result in spurious results that can be misleading about a species’ realized dispersal. Future research needs to focus on the amount of data required to reliably identify IBD when it is present. Our novel SNP data indicate that high throughput sequencing approaches may be suitable to resolve the problem of discrepancies between mtDNA and nuclear markers evident in numerous previous studies. The presence of significant positive relationships between genetic and geographic distance in species where no such relationship was evident for mtDNA data was particularly clear for the neutral SNP data, where the power to correctly detect IBD was maximal and the probability of a type II error was zero. However, given the costs that are presently involved in generating and analysing genome-wide SNP data, this type of marker could be seen as a standard against which less informative but more affordable genetic markers could be measured. We conclude that mtDNA data can be problematic when testing for IBD, as they often do not provide reliable information on a species’ true dispersal potential.

## Electronic supplementary material

Supplementary Information
