# Supplementary material for: Mitochondrial DNA is unsuitable to test for isolation by distance
Source: Sci Rep. 2018 May 31;8:8448. doi: 10.1038/s41598-018-25138-9 (PMC5981212; doi:10.1038/s41598-018-25138-9)
Supplement: Supplementary file 1 — Supplementary Information [file 41598_2018_25138_MOESM1_ESM.docx]

**Mitochondrial DNA is unsuitable to test for isolation by distance**

**Peter R. Teske^1,^* ^,^**, Tirupathi Rao Golla^1,^**, Jonathan Sandoval-Castillo^2^, Arsalan Emami-Khoyi^1^, Carl D. van der Lingen^3^, Sophie von der Heyden^4^, Brent Chiazzari^5^, Bettine Jansen van Vuuren^1^, Luciano B. Beheregaray^2^**

^1^Centre for Ecological Genomics and Wildlife Conservation, Department of Zoology, University of Johannesburg, Auckland Park 2006, South Africa

^2^Molecular Ecology Lab, College of Science and Engineering, Flinders University, Adelaide, SA 5001, Australia

^3^Branch: Fisheries Management, Department of Agriculture, Forestry and Fisheries, Private Bag X2, Vlaeberg 8012, South Africa; and Department of Biological Sciences and Marine Research Institute, University of Cape Town, Private Bag X3, Rondebosch 7700, South Africa

^4^Evolutionary Genomics Group, Department of Botany and Zoology, University of Stellenbosch, Private Bag X1, Matieland 7602

^5^School of Life Sciences, University of KwaZulu-Natal, Westville, Durban, 4001, South Africa

*Corresponding author email: [pteske101@gmail.com](mailto:pteske101@gmail.com)

**These authors contributed equally to this work

Table S1. GPS coordinates of sampling sites in South Africa and South Australia

| **Species** | **Latitude and Longitude** | **No. individuals** | |
| --- | --- | --- | --- |
|  |  | **mtDNA** | **SNP/μsat** |
| ***Sardinops sagax*** | 31° 27' 16"S, 17° 27' 14" E |  | 2 |
|  | 32° 7' 12"S, 18° 16' 12" E |  | 1 |
|  | -33° 35' 21" S, 17° 42' 29.4" E | 13 | 7 |
|  | 33° 35' 27" S, 18° 0' 4" E |  | 6 |
|  | 33° 44' 42" S, 18° 15' 30" E |  | 1 |
|  | 34° 27' 44" S, 18° 22' 49" E |  | 3 |
|  | 34° 28' 6" S, 18° 20' 29" E |  | 1 |
|  | 34° 37' 37" S, 18° 30' 12" E |  | 6 |
|  | 34° 38' 14" S, 18° 148' 16" E |  | 3 |
|  | 34° 27' 18" S, 18° 58' 8" E |  | 9 |
|  | 34° 39' 54" S, 18° 55' 12" E |  | 9 |
|  | 34° 47' 58" S, 18° 50' 34" E |  | 9 |
|  | 34° 33' 19" S, 19° 19' 19" E |  | 1 |
|  | 34° 56' 24" S, 18° 22' 49" E |  | 5 |
|  | 35° 15' 19" S, 20° 10' 8" E |  | 4 |
|  | 35° 48' 16" S, 20° 28' 36" E |  | 3 |
|  | 35° 26' 8" S, 21° 14' 14" E |  | 4 |
|  | 34° 30' 35" S, 2°1 15' 13" E |  | 6 |
|  | 34° 50' 5" S, 22° 14' 16" E |  | 8 |
|  | 34° 34' 20" S, 23° 31' 5" E |  | 10 |
|  | 34° 34' 11"S, 21° 31' 33" E |  | 7 |
|  | 35° 37' 36"S, 21° 8' 31" E |  | 1 |
|  | -34° 37' 36.5"S, 18° 30' 11" E | 6 | 3 |
|  | 34° 37' 17"S, 21° 2' 31" E |  | 7 |
|  | 35° 0' 20" S, 21° 39' 28" E |  | 5 |
|  | 34 23' 18" S, 22 21' 2" E |  | 4 |
|  | 34°42' 13" S, 22°42' 22" E |  | 3 |
|  | 34° 37' 40" S, 22° 21' 13" E |  | 2 |
|  | 34° 4' 9" S, 23° 38' 13" E |  | 1 |
|  | 34° 5' 31" S, 23° 51' 27" E |  | 3 |
|  | 34° 32' 14" S, 24° 25' 27" E |  | 3 |
|  | 34° 16' 1" S, 24° 44' 14" E |  | 5 |
|  | 34° 7' 2" S, 25° 48' 30" E |  | 3 |
|  | 33° 41' 33" S, 26° 51' 11" E |  | 2 |
|  | 33° 55' 21" S, 25° 50' 46" E |  | 3 |
|  | 34° 29' 7" S, 25° 27' 1" E |  | 10 |
|  | 34° 12' 5" S, 26° 0' 29" E |  | 7 |
|  | 33° 30' 7" S, 27° 11' 9" E |  | 10 |
|  | 32° 46' 7" S, 28° 9' 25" E |  | 6 |
|  | -30° 26' 59." S, 29° 48' 59.76" E | 10 | 23 |
|  | -31° 1' 58.8" S, 29° 33' 59.76"E | 13 | 24 |
| ***Psammogobius knysnaensis*** | 33°6'51.43"S, 18°2'44.84"E | 21 | 23 |
|  | 34°17'53.48"S, 18°49'11.10"E | 5 | 4 |
|  | 34°19'52.36S, 18° 59'26.36E |  | 23 |
|  | 34°25'8.06"S, 19°18'23.78"E | 8 | 4 |
|  | 34°24'S, 20°50'E |  | 11 |
|  | 34°36'12.35"S 19°24'52.91"E | 2 |  |
|  | 34°5'29.82"S, 22°8'45.12"E | 19 | 6 |
|  | 33°52'9.06"S, 25°37'43.13"E | 8 | 6 |
|  | 33°40'52.72"S, 26°39'27.80"E | 6 |  |
|  | 31°59'2.81"S, 29°9'0.83"E | 10 | 6 |
|  | 31°39'2.05"S, 29°30'59.14"E | 13 | 26 |
| ***Nerita atramentosa*** | 35°02'15"S, 116°44'40"E | 4 | 4 |
|  | 35°01'48"S, 117°52'56"E | 5 | 5 |
|  | 33°51'38"S, 121°53'46"E | 5 | 5 |
|  | 34°29'22"S, 136°04'39"E | 4 | 4 |
|  | 34°53'44"S, 137°14'59"E | 5 | 5 |
|  | 34°58'50"S, 138°30'35"E | 5 | 5 |
|  | 35°33'01"S, 138°37'34"E | 5 | 5 |
|  | 38°46'50"S, 143°39'59"E | 6 | 6 |
|  | 38°51'39"S, 146°00'14"E | 4 | 4 |
|  | 38°40'04"S, 146°41'60"E | 6 | 6 |
| ***Siphonaria diemenensis*** | 34°08'30"S, 135°14'49"E | 5 | 5 |
|  | 34°29'22"S, 136°04'39"E | 5 | 5 |
|  | 34°53'44"S, 137°14'59"E | 5 | 5 |
|  | 34°58'50"S, 138°30'35"E | 5 | 5 |
|  | 35°33'01"S, 138°37'34"E | 5 | 5 |
|  | 38°46'50"S, 143°39'59"E | 5 | 5 |
|  | 38°51'39"S, 146°00'14"E | 4 | 4 |
|  | 38°40'04"S, 146°41'60"E | 5 | 5 |

| Table S2. Tests for isolation by distance in (a) South African and (b) temperate southern Australian high-dispersal marine species; data originated either from (M) multiple or (S) single marine bioregions (a question mark indicates that this is uncertain, e.g. because IBD is found across a marine bioregion proper and a biogeographical transition area). | | | | | | |
| --- | --- | --- | --- | --- | --- | --- |
| **M/S** | **Species** | **Region** | **Marker** | **Statistic** | **P** | **Reference** |
| 1. South Africa | | | | | | |
| M | *Acanthochiton garnoti* | W, S, E | COI | 0.0015 | **<0.05** | 1 |
| S | " | separate | COI | n/a | >0.05 | " |
| M | *Caffrogobius caffer* | W, S, E | CR | 0.72 | >0.05 | 2 |
| M | *Chrysoblephus laticeps* | W, S, SE | CR | 0.11 | 0.30 | 3 |
|  | " | W, S, SE | μsat | 0.28 | 0.25 | " |
| M | *Cyclograpsus punctatus* | SW, S, E | COI | 0.1279 | **<0.05** | 1 |
| S | " | separate | COI | n/a | >0.05 | " |
| S | *Hymenosoma longicrure* | SE | COI  ANT | 0.001  0.0002 | 0.100  0.106 | 4 |
| S | *H. orbiculare* | S | COI | 0.84 | 0.99 | 5 |
| S | " | E | COI | -0.42 | 0.15 | " |
| M | *Oxystele tigrina* | W, S, E | COI | -0.00112 | **<0.05** | 1 |
| S | " | separate | COI | n/a | >0.05 | " |
| M | *Oxystele variegata* | W, S, E | COI | 0.1149 | **<0.05** | " |
| S | " | separate | COI | n/a | >0.05 | " |
| M | *Mustelus mustelus* | W, S, E | ND4 | 0.0405 | 0.185 | 6 |
| M | " | " | μsat | 0.115 | 0.162 | " |
| S? | *Palaemon capensis* | S, SE | COI | 0.391 | **<0.05** | 7 |
| M | *Palaemon peringueyi* | W, SW, S, SE | COI | 0.163 | **<0.05** | " |
| M | *Parechinus angulosus* | W, S, E | COI | 0.58 | **<0.05** | 8 |
| S | " | separate | COI | n/a | **<0.05** | 1 |
| M | *Perna perna* | W, S, E |  | −0.141 | 0.09 | 9 |
| M | " | W, S | COI | 0.202 | 0.833 | " |
| S | " | E | COI | 0.192 | 0.8 | " |
| M | *Psammogobius knysnaensis* | W, S, E | COI | −0.18 | 0.88 | 10 |
| S | *Sousa sp.* | WIO | CR | 0.001 | 0.536 | 11 |
| M | *Tetraclita serrata* | W, S, E | COI | 8.925 | **<0.05** | 12 |
| S | " | W | COI | 0.119 | >0.05 | " |
| S | " | S | COI | −0.035 | >0.05 | " |
| S | " | E | COI | −0.096 | >0.05 | " |
| M | *Upogebia africana* | W, S | COI | −0.07 | 0.19 | 13 |
| S | " | E | COI | 0.36 | 0.96 | " |
| S |  | E | COI | 0.0007 | 0.33 | 14 |
| (b) temperate Australia | | | | | | |
| M | *Argyrosomus japonicus* | W, S, E | μsat | 0.588 | **0.003** | 15 |
| M | " | S, E | μsat | 0.611 | **<0.001** | " |
| S | *Centrostephanus rodgersii* | E | μsat | −0.07 | <0.01 | 16 |
| S | *Delphinus delphis* | S | μsat | n/a | 0.081 | 17 |
| S | " | E | CR | 0.5697 | 0.14 | 18 |
| S | " | E | μsat | 0.1773 | 0.3451 | " |
| M | *Donax deltoides* | S, E | μsat | 0.70 | **<0.01** | 19 |
| S | " | S | μsat | −0.49 | 0.882 | " |
| S | " | E | μsat | 0.76 | 0.13 | " |
| M | *Haliotis rubra* | S, E | μsat | 0.42 | **<0.05** | 20 |
| M | " | S, E | μsat | 0.20 | **<0.01** | 21 |
| S | " | S | μsat | 0.07 | >0.05 | " |
| M | *Heliocidarus* *erythrogramma* | S, E | μsat | 0.1 | 0.393 | 22 |
| M | *Heliocidaris erythrogramma armigera* | S, E | μsat | 0.25 | 0.063 | " |
| M? | *Octopus maorum* | S, T | μsat | n/a | 0.83 | 23 |
| S | *Pyura praeputialis* | E | COI  ANT | 0.025  −0.060 | 0.234  0.099 | 24 |
| S | " | SE | COI  ANT | −0.109  0.002 | <0.01  0.435 | " |
| S | *Tursiops* sp. | S | μsat | n/a | 0.073 | 17 |
| ANT: intron of the Adenine Nucleotide Transporter (or ADP/ATP translocase) gene; COI: cytochrome *c* oxidase subunit I gene; CR: control region; μsat: microsatellites; W: West, SW: South-west, S: South, SE: South-east, E: East, T: Tasmania, WIO: Western Indian Ocean; in Australia, Wilson’s Promontory was considered the boundary between S and E. Statistic: various Mantel test or regression statistics were reported; we report whether the correlation with geographic distance was positive (i.e. IBD if significant) or negative (no IBD if significant); where multiple statistics were reported whose results were the same, only the first is reported. | | | | | | |

| Table S3. Comparisons of tests for IBD in studies that have tested for it using different types of markers. Significant P-values are shown in bold. | | | | |
| --- | --- | --- | --- | --- |
| **Species** | **Marker** | **Test statistics** | **P** | **Reference** |
| *Alopex lagopus* | mtDNA  Microsatellites | 0.573  0.472 | **<0.001**  **<0.001** | 25 |
| *Anguilla japonica* | mtDNA  Microsatellites | -0.088  0.267 | 0.05  0.05 | 26 |
| *Aphis gossypii* | mtDNA  Microsatellites | 0.357  0.315 | **0.033**  **0.006** | 27 |
| *Atrina pectinata* | mtDNA  Microsatellites | Not  reported | >0.05  >0.05 | 28 |
| *Aythya fuligula* | mtDNA  Microsatellites | 0.003  0.014 | 0.35  0.26 | 29 |
| *Carcharhinus limbatus* | mtDNA  Microsatellites | 0.474  0.259 | **0.0001**  **0.0026** | 30 |
| *Chaetodon meyeri* | mtDNA  Microsatellites | 0.01  −0.09 | 0.37  0.60 | 31 |
| *​Chaetodon ornatissimus* | mtDNA  Microsatellites | 0.30  0.01 | 0.06  0.38 | 31 |
| *Cryptasterina pentagona* | mtDNA  Microsatellites | 0.5025  0.02 | **<0.0459**  0.25 | 32 |
| *Cynomys ludovicianus* | mtDNA  Microsatellites | 0.388  0.839 | 0.067  **0.006** | 33 |
| *Cynomys mexicanus* | mtDNA  Microsatellites | 0.170  −0.05 | 0.274  0.548 | 33 |
| *Cyprinodon macularius* | mtDNA  Microsatellites | Not  reported | 0.26  **0.0001** | 34 |
| *Locustella luscinioides* | mtDNA  Microsatellites | 0.325  0.366 | 0.06  **0.001** | 35 |
| *Lynx rufus fasciatus* and *L. rufus pallescens* | mtDNA  Microsatellites | 0.433  0.422 | **0.005**  **0.002** | 36 |
| *Macrobrachium rosenbergii* | mtDNA  Microsatellites | 0.053  0.192 | 0.400  0.165 | 37 |
| *Micropterus dolomieu* | mtDNA  Microsatellites | 0·05  0.1 | **0.001**  **0.01** | 38 |
| *Microtus californicus* | mtDNA  Microsatellites | Not  reported | 0.340  **0.003** | 39 |
| *Mirounga leonine* | mtDNA  Microsatellites | Not  reported | 0.1039  **0.0296** | 40 |
| *Mugil cephalus* | mtDNA  Microsatellites | 790.27  21.55 | 0.302  **0.015** | 41 |
| *Mustelus mustelus* | mtDNA  Microsatellites | 0.0405  0.1152 | 0.185  0.162 | 6 |
| *Myotis pilosus* | mtDNA  Microsatellites | 0.21  0.56 | 0.05  **0.001** | 42 |
| *Myripristis berndti* | mtDNA  Microsatellites | 0.35  0.46 | 0.12  **0.01** | 43 |
| *Orchestia montagui* | mtDNA  Microsatellites | −0.000038  −0.186 | 0.807  0.633 | 44 |
| *Panulirus interruptus*  (All sites) | mtDNA  Microsatellites | 0.012  0.011 | 0.154  0.177 | 45 |
| *Panulirus interruptus*  (Island sites) | mtDNA  Microsatellites | 0.258  0.093 | **0.022**  0.111 | 45 |
| *Pomatoschistus minutus* | mtDNA  Microsatellites | 0.6804  0.8574 | **0.032**  **0.001** | 46 |
| *Pontoporia blainvillei* | mtDNA  Microsatellites | 0.337  0.281 | **0.01**  0.1 | 47 |
| *Pontorporia blainvillei* | mtDNA  Microsatellites | 0.128  0.325 | 0.373  0.333 | 48 |
| *Pristipomoides filamentosus*  (all sites) | mtDNA  Microsatellites | 0.28  Not reported | **0.012**  No IBD | 49 |
| *Pristipomoides filamentosus* (excluding Salmon Banks) | mtDNA  Microsatellites | 0.06  Not  reported | 0.244  No IBD | 49 |
| *Rhinolophus monoceros*  (both sexes) | mtDNA  Microsatellites | 0.816  0.450 | **<0.001**  **<0.001** | 50 |
| *Rhinolophus monoceros*  (females) | mtDNA  Microsatellites | 0.769  0.096 | **<0.001**  **<0.001** | 50 |
| *Rhinolophus monoceros*  (males) | mtDNA  Microsatellites | 0.850  0.427 | **<0.01**  **<0.05** | 50 |
| *Semibalanus balanoides* | mtDNA  Microsatellites | Not  reported | 0.4245  **0.0001** | 51 |
| *Serrasalmus rhombeus* | mtDNA  Microsatellites | 81.76  11.27 | **0.04**  **0.0005** | 52 |
| *Sigaus australis* | mtDNA  Microsatellites | Not  reported | 0.5410  **0.0108** | 53 |
| *Somateria mollissima* | mtDNA  Microsatellites | 0.255  0.642 | 0.091  **0.001** | 54 |
| *Sorex antinorii* | mtDNA  Microsatellites | 0.188  0.333 | **0.001**  **0.001** | 55 |
| *Spheniscus magellanicus* | mtDNA  Microsatellites | 0.218  0.207 | 0.093  0.098 | 56 |
| *Sprattus sprattus* | mtDNA  Microsatellites | 0.89  0.038 | 0.31  **0.01** | 57 |
| *Sternula antillarum*  (all breeding areas) | mtDNA  Microsatellites | 0.394  0.385 | **0.002**  **<0.001** | 58 |
| *S. antillarum*  (interior breeding areas) | mtDNA  Microsatellites | -0.190  0.517 | 0.845  **0.011** | 58 |
| *S. antillarum*  (eastern breeding area) | mtDNA  Microsatellites | -0.007  0.368 | **0.471**  **0.049** | 58 |
| *Strix aluco* | mtDNA  Microsatellites | Not  reported | 0.38  0.86 | 59 |
| *Tetragonisca angustula* | mtDNA  Microsatellites | 0.415  0.464 | **0.004**  **0.001** | 60 |
| *Trematomus bernacchii* | mtDNA  Microsatellites | 0.3109  0.52 | **0.03**  0.054 | 61 |
| *T. bernacchii* (excluding EIa) | mtDNA  Microsatellites | 0.5165  0.52 | **0.01**  **0.01** | 62 |
| *Trematomus hansoni* | mtDNA  Microsatellites | −0.0078  0.60 | 0.49  **0.03** | 62 |
| *Trematomus hansoni* (excl. SGa0) | mtDNA  Microsatellites | −0.1046  0.79 | 0.73  **0.001** | 62 |
| *Trematomus newnesi* | mtDNA  Microsatellites | 0.6814  0.63 | 0.07  **0.02** | 62 |
| *Tridacna crocea* | mtDNA  Microsatellites | 0.5073  0.5883 | 0.332  **0.001** | 63 |
| *Tympanuchus cupido pinnatus*  (all populations) | mtDNA  Microsatellites | 0.26  0.12 | **0.019**  0.075 | 64 |
| *T. cupido pinnatus*  (excluding Wisconsin) | mtDNA  Microsatellites | 0.51  0.04 | **0.041**  0.285 | 64 |
| *T. cupido pinnatus*  (Wisconsin only) | mtDNA  Microsatellites | 0.27  0.35 | 0.792  0.339 | 64 |
| *Zalophus californianus* | mtDNA  Microsatellites | 0.672  0.902 | **0.048**  **0.001** | 65 |

| Table S4. Multiple linear regression analyses of geographic (indepdenent variable) vs. genetic (dependent variable) distance in the four study species. Bold P-values and values indicating the power of the analysis of variance of particular test are both shown for α = 0.05 and indicate significance and a minimum power of at least 0.8, respectively. | | | | | |
| --- | --- | --- | --- | --- | --- |
| Marker | Species | *R^2^* | *F* | P | Power |
| mtDNA COI | *Sardinops sagax* | 0.00 | 0.0 | 0.993 | 0.03 |
|  | *Psammogobius knysnaensis* | 0.00 | 0.1 | 0.811 | 0.04 |
|  | *Nerita atramentosa* | 0.02 | 1.4 | 0.235 | 0.22 |
|  | *Siphonaria diemenensis* | 0.02 | 0.5 | 0.464 | 0.11 |
| SNPs | *Sardinops sagax* | 0.39 | 84.7 | **<0.001** | **1.00** |
|  | *Psammogobius knysnaensis* | 0.81 | 109.6 | **<0.001** | **1.00** |
| Microsatellites | *Nerita atramentosa* | 0.03 | 2.3 | 0.131 | 0.33 |
|  | *Siphonaria diemenensis* | 0.29 | 13.6 | **<0.001** | **0.93** |


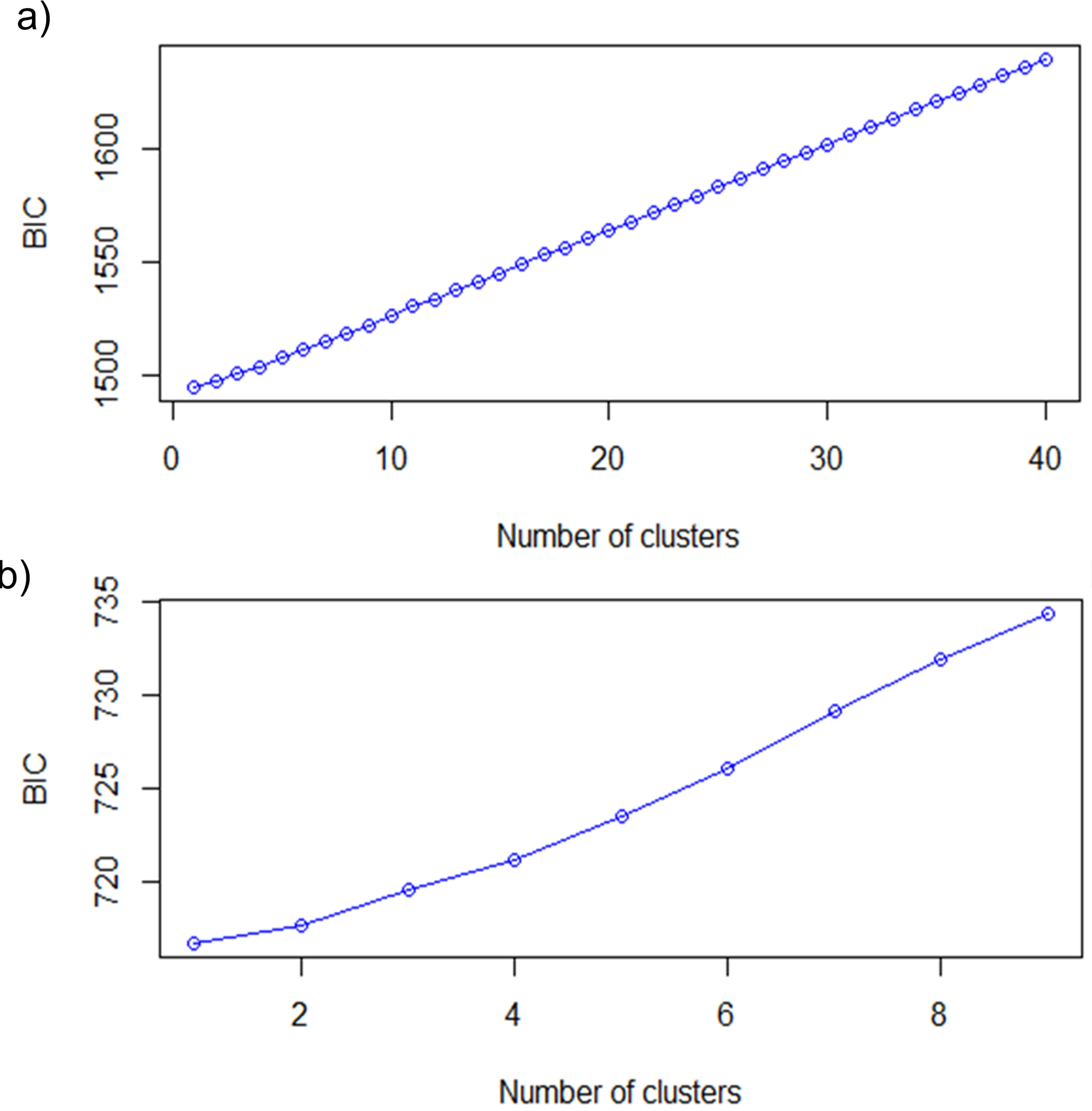


Fig. S1. Bayesian Information Criterion (BIC) plots to identify the number of distinct genetic clusters in the selectively neutral SNP data of a) *Sardinops sagax* and b) *Psammogobius knysnaensis.*

**References**

1. Wright, D., Bishop, J. M., Matthee, C. A. & von der Heyden, S. Genetic isolation by distance reveals restricted dispersal across a range of life histories: implications for biodiversity conservation planning across highly variable marine environments. *Divers. Distrib.* **21,** 698–710 (2015).

2. Neethling, M., Matthee, C. A., Bowie, R. C. K. & von der Heyden, S. Evidence for panmixia despite barriers to gene flow in the southern African endemic, *Caffrogobius caffer* (Teleostei: Gobiidae). *BMC Evol. Biol.* **8,** 325–325 (2008).

3. Teske, P. R., Forget, F. R. G., Cowley, P. D., von der Heyden, S. & Beheregaray, L. B. Connectivity between marine reserves and exploited areas in the philopatric reef fish *Chrysoblephus laticeps* (Teleostei: Sparidae). *Mar. Biol.* **157,** 2029–2042 (2010).

4. Qhaji, Y., Jansen van Vuuren, B., Papadopoulos, I., McQuaid, C. D. & Teske, P. R. A comparison of genetic structure in two low-dispersal crabs from the Wild Coast, South Africa. *Afr. J. Mar. Sci.* **37,** 345–351 (2015).

5. Teske, P. R., Froneman, P. W., Barker, N. P. & McQuaid, C. D. Phylogeographic structure of the caridean shrimp *Palaemon peringueyi* in South Africa: further evidence for intraspecific genetic units associated with marine biogeographic provinces. *Afr. J. Mar. Sci.* **29,** 253–258 (2007).

6. Maduna SN, da Silva C, Wintner SP, Roodt-Wilding R & Bester-van der Merwe AE. When two oceans meet: regional population genetics of an exploited coastal shark, *Mustelus mustelus*. *Mar. Ecol. Prog. Ser.* **544,** 183–196 (2016).

7. Wood, L. E., De Grave, S. & Daniels, S. R. Phylogeographic patterning among two codistributed shrimp species (Crustacea: Decapoda: Palaemonidae) reveals high levels of connectivity across biogeographic regions along the South African coast. *PLOS ONE* **12,** 1–18 (2017).

8. Muller, C., von der Heyden, S., Bowie, R. & Matthee, C. Oceanic circulation, local upwelling and palaeoclimatic changes linked to the phylogeography of the Cape sea urchin *Parechinus angulosus*. *Mar. Ecol. Prog. Ser.* **468,** 203–215 (2012).

9. Zardi, G. I., McQuaid, C. D., Teske, P. R. & Barker, N. P. Unexpected genetic structure of mussel populations in South Africa: indigenous *Perna perna* and invasive *Mytilus galloprovincialis*. *Mar. Ecol. Prog. Ser.* **337,** 135–144 (2007).

10. Drost, E., Golla, T. R., von der Heyden, S. & Teske, P. R. No divergent evolution, despite restricted connectivity, between Atlantic and Indian Ocean goby populations. *Mar. Biodivers.* (2015). doi:10.1007/s12526-015-0389-6

11. Mendez, M. *et al.* Molecular ecology meets remote sensing: environmental drivers to population structure of humpback dolphins in the Western Indian Ocean. *Heredity* **107,** 349 (2011).

12. Reynolds, T. V., Matthee, C. A. & Von Der Heyden, S. The influence of Pleistocene climatic changes and ocean currents on the phylogeography of the southern African barnacle, *Tetraclita serrata* (Thoracica; Cirripedia). *PLoS ONE* **9,** (2014).

13. Teske, P. R., McQuaid, C. D., Froneman, P. W. & Barker, N. P. Impacts of marine biogeographic boundaries on phylogeographic patterns of three South African estuarine crustaceans. *Mar. Ecol. Prog. Ser.* **314,** 283–293 (2006).

14. Teske, P. R. *et al.* Oceanic dispersal barriers, adaptation and larval retention: an interdisciplinary assessment of potential factors maintaining a phylogeographic break between sister lineages of an African prawn. *BMC Evol. Biol.* **8,** 341–341 (2008).

15. Barnes, T. C. *et al.* Population structure in a wide-ranging coastal teleost (*Argyrosomus japonicus*, Sciaenidae) reflects marine biogeography across southern Australia. *Mar. Freshw. Res.* **67,** 1103–1113 (2016).

16. Banks, S. C. *et al.* Oceanic variability and coastal topography shape genetic structure in a long-dispersing sea urchin. *Ecology* **88,** 3055–3064 (2007).

17. Bilgmann, K., Möller, L. M., Harcourt, R. G., Gales, R. & Beheregaray, L. B. Common dolphins subject to fisheries impacts in Southern Australia are genetically differentiated: Implications for conservation. *Anim. Conserv.* **11,** 518–528 (2008).

18. Möller, L. *et al.* Fine-scale genetic structure in short-beaked common dolphins (*Delphinus delphis*) along the East Australian Current. *Mar. Biol.* **158,** 113–126 (2011).

19. Miller, A. D., Versace, V. L., Matthews, T. G., Montgomery, S. & Bowie, K. C. Ocean currents influence the genetic structure of an intertidal mollusc in southeastern Australia - implications for predicting the movement of passive dispersers across a marine biogeographic barrier. *Ecol. Evol.* **3,** 1248–1261 (2013).

20. Huang, B. X., Peakall, R. & Hanna, P. J. Analysis of genetic structure of blacklip abalone (Haliotis rubra) populations using RAPD, minisatellite and microsatellite markers. *Mar. Biol.* **136,** 207–216 (2000).

21. Miller, A. D. *et al.* Contrasting patterns of population connectivity between regions in a commercially important mollusc *Haliotis rubra*: integrating population genetics, genomics and marine LiDAR data. *Mol. Ecol.* **25,** 3845–3864 (2016).

22. Binks, R. M., Evans, J. P., Prince, J. & Kennington, W. J. Fine-scale patterns of genetic divergence within and between morphologically variable subspecies of the sea urchin *Heliocidaris erythrogramma* (Echinometridae). *Biol. J. Linn. Soc.* **103,** 578–592 (2011).

23. Doubleday, Z. A., Semmens, J. M., Smolenski, A. J. & Shaw, P. W. Microsatellite DNA markers and morphometrics reveal a complex population structure in a merobenthic octopus species (*Octopus maorum*) in south-east Australia and New Zealand. *Mar. Biol.* **156,** 1183–1192 (2009).

24. Teske, P. R. Connectivity in solitary ascidians: Is a 24-h propagule duration sufficient to maintain large-scale genetic homogeneity? *Mar. Biol.* **161,** 2681–2687 (2014).

25. Geffen, E. *et al.* Sea ice occurrence predicts genetic isolation in the Arctic fox. *Mol. Ecol.* **16,** 4241–4255 (2007).

26. Gong, X., Ren, S., Cui, Z. & Yue, L. Genetic evidence for panmixia of Japanese eel (*Anguilla japonica*) populations in China. *Genet. Mol. Res. GMR* **13,** 768—781 (2014).

27. Wang, X.-Y., Yang, X.-M., Lu, B., Zhou, L.-H. & Wu, K.-M. Genetic variation and phylogeographic structure of the cotton aphid, *Aphis gossypii*, based on mitochondrial DNA and microsatellite markers. *Sci. Rep.* **7,** 1920 (2017).

28. Xue, D.-X., Wang, H.-Y., Zhang, T. & Liu, J.-X. Population genetic structure and demographic history of *Atrina pectinata* based on mitochondrial DNA and microsatellite Markers. *PLOS ONE* **9,** 1–12 (2014).

29. Liu, Y., Keller, I. & Heckel, G. Breeding site fidelity and winter admixture in a long-distance migrant, the tufted duck (*Aythya fuligula*). *Heredity* **109,** 108–116 (2012).

30. Keeney, D. B., Heupel, M. R., Hueter, R. E. & Heist, E. J. Microsatellite and mitochondrial DNA analyses of the genetic structure of blacktip shark (*Carcharhinus limbatus*) nurseries in the northwestern Atlantic, Gulf of Mexico, and Caribbean Sea. *Mol. Ecol.* **14,** 1911–1923 (2005).

31. Di Battista, J. D., Rocha, L. A., Craig, M. T., Feldheim, K. A. & Bowen, B. W. Phylogeography of two closely related Indo-Pacific butterflyfishes reveals divergent evolutionary histories and discordant results from mtDNA and microsatellites. *J. Hered.* **103,** 617–629 (2012).

32. Puritz, J. B. *et al.* Extraordinarily rapid life-history divergence between cryptasterina sea star species. *Proc. R. Soc. B Biol. Sci.* **279,** 3914 (2012).

33. Castellanos-Morales, G., Gámez, N., Castillo-Gámez, R. A. & Eguiarte, L. E. Peripatric speciation of an endemic species driven by Pleistocene climate change: The case of the Mexican prairie dog (*Cynomys mexicanus*). *Mol. Phylogenet. Evol.* **94,** 171–181 (2016).

34. Loftis, D. G., Echelle, A. A., Koike, H., Van Den Bussche, R. A. & Minckley, C. O. Genetic structure of wild populations of the endangered Desert Pupfish complex (Cyprinodontidae: Cyprinodon). *Conserv. Genet.* **10,** 453–463 (2009).

35. Neto, J. M. *et al.* Phylogeography of a habitat specialist with high dispersal capability: The Savi’s warbler *Locustella luscinioides*. *PLOS ONE* **7,** 1–14 (2012).

36. Reding, D. M., Carter, C. E., Hiller, T. L. & Clark, W. R. Using population genetics for management of bobcats in Oregon. *Wildl. Soc. Bull.* **37,** 342–351 (2013).

37. De Bruyn, M. & Mather, P. B. Molecular signatures of Pleistocene sea-level changes that affected connectivity among freshwater shrimp in Indo-Australian waters. *Mol. Ecol.* **16,** 4295–4307 (2007).

38. Stepien, C. A., Karsiotis, S. I., Sullivan, T. J. & Klymus, K. E. Population genetic structure and comparative diversity of smallmouth bass *Micropterus dolomieu*: congruent patterns from two genomes. *J. Fish Biol.* **90,** 2125–2147 (2017).

39. Adams, R. I. & Hadly, E. A. High levels of gene flow in the California Vole (*Microtus californicus*) are consistent across spatial scales. *West. North Am. Nat.* **70,** 296–311 (2010).

40. Corrigan, L. J. *et al.* Population differentiation in the context of Holocene climate change for a migratory marine species, the southern elephant seal. *J. Evol. Biol.* **29,** 1667–1679 (2016).

41. Durand JD, Blel H, Shen KN, Koutrakis ET & Guinand B. Population genetic structure of *Mugil cephalus* in the Mediterranean and Black Seas: a single mitochondrial clade and many nuclear barriers. *Mar. Ecol. Prog. Ser.* **474,** 243–261 (2013).

42. Lu, G. *et al.* Phylogeography of the Rickett’s big-footed bat, *Myotis pilosus* (Chiroptera: Vespertilionidae): a novel pattern of genetic structure of bats in China. *BMC Evol. Biol.* **13,** 241 (2013).

43. Muths, D., T., E. Gouws, G. Craig, M. Mwale, M. Mwaluma, J. Mwandya, A. Bourjea, J. Restricted dispersal of the reef fish *Myripristis berndti* at the scale of the SW Indian Ocean. **443,** (2011).

44. Pavesi, L., Deidun, A., De Matthaeis, E., Tiedemann, R. & Ketmaier, V. Mitochondrial DNA and microsatellites reveal significant divergence in the beachflea *Orchestia montagui* (Talitridae: Amphipoda). *Aquat. Sci.* **74,** 587–596 (2012).

45. Iacchei, M. *et al.* Combined analyses of kinship and FST suggest potential drivers of chaotic genetic patchiness in high gene-flow populations. *Mol. Ecol.* **22,** 3476–3494 (2013).

46. Larmuseau, M. H. D., Raeymaekers, J. A. M., Hellemans, B., Houdt, J. K. J. V. & Volckaert, F. A. M. Mito-nuclear discordance in the degree of population differentiation in a marine goby. *Heredity* **105,** 532–542 (2010).

47. Mendez, M., Rosenbaum, H. C., Subramaniam, A., Yackulic, C. & Bordino, P. Isolation by environmental distance in mobile marine species: Molecular ecology of franciscana dolphins at their southern range. *Mol. Ecol.* **19,** 2212–2228 (2010).

48. Gariboldi, M. C. *et al.* Patterns of population structure at microsatellite and mitochondrial DNA markers in the franciscana dolphin (*Pontoporia blainvillei*). *Ecol. Evol.* **6,** 8764–8776 (2016).

49. Gaither, M. R. *et al.* High connectivity in the deepwater snapper *Pristipomoides filamentosus* (Lutjanidae) across the Indo-Pacific with isolation of the Hawaiian Archipelago. *PLOS ONE* **6,** 1–13 (2011).

50. Chen, S.-F., Jones, G. & Rossiter, S. J. Sex-biased gene flow and colonization in the Formosan lesser horseshoe bat: inference from nuclear and mitochondrial markers. *J. Zool.* **274,** 207–215 (2008).

51. Flight, P. A., O’Brien, M. A., Schmidt, P. S. & Rand, D. M. Genetic structure and the North American postglacial expansion of the barnacle, *Semibalanus balanoides*. *J. Hered.* **103,** 153–165 (2012).

52. Hubert, N. *et al.* Isolation by distance and Pleistocene expansion of the lowland populations of the white piranha *Serrasalmus rhombeus*. *Mol. Ecol.* **16,** 2488–2503 (2007).

53. Dowle, E. J., Morgan-Richards, M. & Trewick, S. A. Morphological differentiation despite gene flow in an endangered grasshopper. *BMC Evol. Biol.* **14,** 216 (2014).

54. Tiedemann, R. *et al.* Mitochondrial DNA and microsatellite variation in the eider duck (*Somateria mollissima*) indicate stepwise postglacial colonization of Europe and limited current long-distance dispersal. *Mol. Ecol.* **13,** 1481–1494 (2004).

55. Yannic, G., Basset, P., Büchi, L., Hausser, J. & Broquet, T. Scale-specific sex-biased dispersal in the *Valais shrew* unveiled by genetic variation on the Y chromosome, autosomes, and mitochondrial DNA. *Evolution* **66,** 1737–1750 (2012).

56. Bouzat, J. L., Walker, B. G. & Boersma, P. D. Regional Genetic Structure in the Magellanic Penguin (*Spheniscus magellanicus*) Suggests Metapopulation Dynamics. *The Auk* **126,** 326–334 (2009).

57. Limborg, M. T. *et al.* Imprints from genetic drift and mutation imply relative divergence times across marine transition zones in a pan-European small pelagic fish (*Sprattus sprattus*). *Heredity* **109,** 96–107 (2012).

58. Draheim, H. M., Miller, M. P., Baird, P. & Haig, S. M. Subspecific status and population genetic structure of least terns (*Sternula antillarum*) inferred by mitochondrial DNA control-region sequences and microsatellite DNA. *The Auk* **127,** 807–819 (2010).

59. Brito, P. H. Contrasting patterns of mitochondrial and microsatellite genetic structure among Western European populations of tawny owls (*Strix aluco*). *Mol. Ecol.* **16,** 3423–3437 (2007).

60. Francisco, F. O., Santiago, L. R., Mizusawa, Y. M., Oldroyd, B. P. & Arias, M. C. Population structuring of the ubiquitous stingless bee *Tetragonisca angustula* in southern Brazil as revealed by microsatellite and mitochondrial markers. *Insect Sci.* **24,** 877–890 (2017).

61. Putte, A. P. V. de *et al.* Comparative phylogeography of three trematomid fishes reveals contrasting genetic structure patterns in benthic and pelagic species. *Mar. Genomics* **8,** 23–34 (2012).

62. Putte, A. P. V. de *et al.* Comparative phylogeography of three trematomid fishes reveals contrasting genetic structure patterns in benthic and pelagic species. *Mar. Genomics* **8,** 23–34 (2012).

63. DeBoer, T. S. *et al.* Concordant phylogenetic patterns inferred from mitochondrial and microsatellite DNA in the giant clam *Tridacna crocea.* *Bull. Mar. Sci.* **90,** 301–329 (2014).

64. Johnson, J. A., Toepfer, J. E. & Dunn, P. O. Contrasting patterns of mitochondrial and microsatellite population structure in fragmented populations of greater prairie-chickens. *Mol. Ecol.* **12,** 3335–3347 (2003).

65. Gonzalez-Suarez, M., Flatz, R., Aurioles-Gamboa, D., Hedrick, P. W. & Gerber, L. R. Isolation by distance among California sea lion populations in Mexico: redefining management stocks. *Mol. Ecol.* **18,** 1088–1099 (2009).
